# Paramedics Providing Palliative Care at Home: A Retrospective Cohort Study Comparing Symptom Management of Breathlessness and Pain in Cancer Versus Non-Cancer Conditions

**DOI:** 10.7759/cureus.64750

**Published:** 2024-07-17

**Authors:** Brianne Robinson, Judah Goldstein, Michelle Harrison, Marianne Arab, Alix Carter

**Affiliations:** 1 Emergency Medicine, Dalhousie University, Halifax, CAN; 2 Cancer Care Program, Nova Scotia Health, Halifax, CAN

**Keywords:** breathlessness, pain, prehospital, paramedics, end-of-life, palliative

## Abstract

Background

Palliative care aims to alleviate pain and distressing symptoms, affirm life, and offer support to patients and their caregivers. For many, the expressed preference is to die at home. As a result, there is growing recognition that paramedics can play an integral role at the end of life for symptom relief. Paramedic comfort with symptom management in the palliative care context is suspected, based on past work, to be higher for cancer as opposed to non-cancer life advanced disease. The objective of this study was to explore the paramedic management of patients with cancer and non-cancer advanced disease, using pain and breathlessness as key symptoms.

Methods

A retrospective cohort study was conducted. Paramedic electronic patient care records were queried for calls with palliative goals of care between July 1, 2015, and June 30, 2016, in Nova Scotia, Canada, which was the first year of the Paramedics Providing Palliative Care program. A manual chart review of a subgroup of 100 consecutive charts was completed to gain deeper insight. A descriptive analysis was conducted to understand practice variation within this population.

Results

The electronic query returned 1909 calls with a palliative approach. A total of 765 (40.1%) had cancer. The most common non-cancer disease category was respiratory. The top chief complaint was respiratory distress in both cancer and non-cancer populations. Medication was administered more often for pain (80%) compared to breathlessness (46.5%). Paramedics were more likely to call Medical Oversight Physicians for pain control advice. Post-treatment pain scores were documented infrequently. In the chart review, symptom management using the patient’s own medications occurred in 17% of cases while an additional 5% of cases involved a combination of the patient’s medications and paramedic service formulary.

Conclusion

The non-cancer population was less likely to have a non-transport outcome. Opportunities for improvement of symptom management were noted for pain and particularly so for breathlessness. Increased comfort with a palliative approach in the non-cancer disease cohort as well as with this key symptom will be a key to the success of the program.

## Introduction

Palliative care aims to alleviate pain and distress while offering support to patients with life-limiting illnesses and their families. While efforts are made to control and minimize symptoms, those receiving palliative care may still encounter sudden distressing symptoms which may be physical and/or emotional/psychosocial in nature [1]. Pain and breathlessness tend to be common symptoms requiring support for patients receiving palliative care [2], and an avoidable visit to the emergency department (ED) for almost a quarter of patients [3], resulting in admissions over half of the time [4]. However, a visit to the ED can be an indicator of lower quality end-of-life care [5] as the ED is not considered the ideal environment for those receiving palliative care [6,7]. Nevertheless, patients and families report feeling unprepared to manage acute urgent symptoms at home [8].

There is a growing recognition that care provided by paramedics plays an integral role in the relief of unexpected symptoms experienced by patients with palliative care needs in the community [2,9]. Paramedics are often called for pain and symptom management but traditional protocols fail to address some significant needs of this population, such as the desire to remain at home [10]. In 2015, Nova Scotia implemented the innovative Paramedics Providing Palliative Care at Home program across the full ground ambulance service. The core components include a palliative care-focused clinical practice guideline and paramedic-specific palliative care training enabling paramedics to assist patients and families during symptom crises (e.g., breathlessness, nausea, pain, psycho-social support, administration of opioids and other medications, etc.), without having to take the patient to the hospital ED if medically appropriate and desired by the patient/family. In previous research, patients/families reported high satisfaction with the palliative care emergency support provided at home by paramedics including the symptom relief that was delivered [11].

A palliative approach is not just beneficial to those with life-limiting illnesses such as cancer but also for those with non-cancer advanced diseases such as congestive heart failure (CHF), chronic obstructive pulmonary disease (COPD), frailty, and dementia [12]. Even so, palliative care tends to be initiated later in the disease trajectory for non-cancer advanced diseases [13]. While paramedic practice already includes the administration of medications for many of these indications, in the same drug classes and via the same modalities, how this extends to the context of palliative care is not well understood particularly for non-cancer advanced disease. The rationale for the present study stems from our past work showing that paramedics are less comfortable managing non-cancer advanced diseases with a palliative approach as compared with cancer, and less comfortable managing breathlessness compared with other symptoms like pain or nausea [11].

Pain and breathlessness are the most common reasons for paramedic calls in these patient groups. Breathlessness is also treated differently under a palliative approach using opioids as opposed to usual care, which presents an additional challenge to paramedics' level of comfort in optimally managing this symptom [11]. Routine quality assurance and many research projects rely on data available through electronic queries rather than the far more labour-intensive manual chart review, but we suspected there might be additional information in the free text portions of the chart about practices such as support with patient's own home medications, and this practice of relying on electronic data may give a false impression of the care actually provided. Our objective was to describe symptom management and transport outcomes in cancer versus non-cancer advanced diseases, looking specifically at the common symptoms of pain and breathlessness.

## Materials and methods

Study design

This was a retrospective cohort study. The paramedic electronic patient care record (ePCR) was queried for all patients with palliative goals of care from July 1, 2015, to June 30, 2016, using patient definitions defined below under "participants." In a sub-analysis, a manual chart review of the 100 most recent consecutive paramedic palliative care responses between February 1, 2016, to June 30, 2016, was completed to look in depth at the care provided, through details that would not be captured in an electronic data query such as free text documentation of support with patient's home medications. Data are reported in accordance with the Strengthening the Reporting of Observational Studies in Epidemiology (STROBE) guideline (see Appendices) [12]. This study was approved by the Nova Scotia Health Research Ethics Board (ROMEO No.1021860).

Setting

The study was conducted in the province of Nova Scotia, Canada, with a population of just under one million people. Emergency Health Services (EHS) is the sole emergency medical services (EMS)/paramedic services provider for the province, with a mix of advanced care paramedics (ACP) and primary care paramedics (PCP) responding to approximately 70,000 calls annually during the study period. PCPs account for the largest portion of registered paramedics and have a wide range of scope including administering medications and intravenous access. The ACP scope builds upon the PCP scope further to include advanced techniques such as intubation and administering a wider range of medications such as opioids.

Participants

The study population consisted of all patients with an EMS response classified as a “probable palliative care call.” The study team classified all calls into "probable palliative" or "probable not palliative" using a previously validated algorithm (using a combination of factors such as medical history, chief complaint, and documentation of a Do Not Resuscitate order) to query the ePCR data to select calls that had a high likelihood of being consistent with a paramedic palliative approach to care [13]. We excluded interfacility transfers, critical care transport, “No patient found” selected as ePCR response outcome, those in long-term care, or patients treated within a collaborative emergency centre (urgent care facility staffed by a paramedic and nurse).

Data sources

The EHS Special Patient Program (SPP) database houses patient goals of care which are made available to responding paramedics. The SPP was also queried to obtain a primary diagnosis in order to classify patients as cancer or non-cancer supplemented by past medical history in ePCR if necessary. The ePCR contains data pertaining to patient characteristics, paramedic service operations (e.g. time intervals), and treatments provided.

Statistical methods

Descriptive analysis was conducted, and results were compared with a t-test and Bonferroni correction (statistical significance set at p<0.007). Results were reported descriptively for the overall population and compared between the cancer and non-cancer groups with n and %, median and interquartile range (IQR) or mean and standard deviation (SD) as appropriate. The primary outcome was percent treatment by paramedics while secondary outcomes included transport disposition and treatment efficacy. Both primary and secondary outcomes were evaluated and compared between the main grouping of cancer versus non-cancer as well as a sub-group analysis by pain versus breathlessness. This included total opioid administered, use of home medications, and use of medications from paramedic service formulary.

## Results

In the electronic data query, a total of 1909 ground ambulance responses met the definition of "probable palliative call" and met the inclusion criteria; these comprised 995 unique patients. These responses were divided by the two groups of interest, namely cancer and non-cancer advanced disease; 40.1% (n=765) were cancer and 59.9% (n=1144) were non-cancer. The patient population for the entire electronic query is described in Table 1.

**Table 1:** Patient characteristics *n=13 coded as Other, primary palliative diagnosis missing in 937 as some patients may not have been enrolled in the SPP SD: standard deviation; SPP: Special Patient Program

	Cancer (n=765)	Non-cancer advanced disease (n=1144)
Sex female, n (%)	357 (46.7)	538 (47.0)
Age (years), mean±SD	73.3±11.6	77.7±12.8
Comorbidity count (range)	0-3	0-5
Top five chief complaints, n (%)	Respiratory distress: 83 (10.8); Weakness/fatigue: 83 (10.8); General malaise: 76 (9.9); Other: 75 (9.8); Wellness check: 69 (9.0)	Respiratory distress: 246 (21.5); Wellness check: 113 (9.9); General malaise: 93 (8.1); Weakness/fatigue: 86 (7.5); Other 84 (7.3)
Primary palliative diagnosis, n (%)	Cancer: 765 (100)	Respiratory: 84 (7.3); Dementia: 20 (1.7); Nephrology: 18 (1.6); Cardiac: 42 (3.7); Frailty: 16 (1.4); Neurology: 14 (1.2); *Other/palliative: 950 (83.0)

Similar transport dispositions were observed among the cohorts (Table 2).

**Table 2:** Transport outcome CEC: Collaborative Emergency Centre

	Cancer (n=765), n (%)	Non-Cancer (n=1144), n (%)
Transported	537 (70.2)	792 (69.2)
Non-Transport	186 (24.3)	271 (23.7)
Other (Death, Transfer, CEC, Community Paramedicine)	42 (5.5)	81 (7.1)

Overall, no differences were found in how often Medical Oversight Physicians were consulted in cancer versus non-cancer subgroups, 11.5% vs 10.1%, p=0.35. We looked specifically at all the medications in the paramedic formulary for pain, breathlessness, and nausea; some differences in individual therapies were notable and are presented in Table 3.

**Table 3:** Comparison of treatments in cancer vs non-cancer NS: not significant; n/a: not applicable

	Cancer (n=765), n (%)	Non-Cancer (n =1144), n (%)	P value
Morphine Sulphate	83 (10.8)	55 (4.8)	p<0.001
Hydromorphone	9 (1.2)	<5	p=0.014
Fentanyl	10 (1.3)	6 (0.5)	NS
Midazolam	5 (0.7)	6 (0.5)	NS
Metoclopramide	39 (5.1)	8 (0.7)	NS
Dimenhydrinate	34 (4.4)	19 (1.7)	NS
Haloperidol	<5	32 (2.8)	NS
Diazepam	<5	7 (0.6)	NS
Salbutamol	38 (5.0)	5 (0.4)	p<0.001
Ipratropium Bromide	27 (3.5)	134 (11.7)	p<0.001
Furosemide	0	113 (9.9)	n/a

When comparing the chief complaints of pain and breathlessness, treatment for breathlessness continues to be by metered dose inhalers as opposed to opioids (Table 4). Post-treatment pain scores were documented infrequently (documented in cancer patients 58.7% pre-treatment vs 25.6% post-treatment (p<0.001), in non-cancer 57.4% vs 26.9% (p<0.001)).

**Table 4:** Comparison of treatments by chief complaints of pain or breathlessness. ^*^included abdominal pain, back pain, headache, and chest pain; ^**^included respiratory distress and arrest NS: not significant; n/a: not applicable

	Pain^*^ (n=252), n (%)	Breathlessness^**^ (n=330), n (%)	P value
Morphine Sulphate	53 (21)	14 (4.2)	p<0.001
Hydromorphone	<5	0	NS
Fentanyl	9 (3.6)	0	NS
Midazolam	0	<5	NS
Metoclopramide	11 (4.4)	<5	NS
Dimenhydrinate	21 (8.3)	<5	NS
Haloperidol	0	<5	NS
Diazepam	<5	0	NS
Salbutamol	5 (2)	140 (42.4)	p<0.001
Ipratropium Bromide	<5	115 (34.8)	p<0.001
Furosemide	0	0	n/a

The manual chart review included 100 consecutive unique patients, of whom 94 had one call and six patients had between two and four calls. The chief complaint for 45 calls was pain, and for 33 calls it was breathlessness (our two chief complaints of interest). Including any medication from both paramedics or the patient’s own stock, some type of medication was given in 58 of the calls; 70% of those calls remained at home. Non-transport decreased by 42% when no medication was given (Table 5).

**Table 5:** Transport disposition by treatment status of patients included in the chart review (N=100)

	Transported (n=41)	Not transported (n=59)
Treated, n (%)	16 (39.0%)	42 (71.2%)
No treatment given, n (%)	25 (61.0%)	17 (28.8%)

Of those included in the chart review, 80% of patients with pain and 46.4% of patients with breathlessness received medication. In the 58 cases where medication was given, 78% was from paramedic stock, 17% from patients’ medication only, and 5% was both. Patients' own medication was documented in the narrative only and would be undetected in an electronic query. The median opioid dose given from paramedic stock (in morphine equivalents) was 10 mg (IQR 10-20). A median of 8.75 mg (IQR 5.44-11.25) morphine equivalent was given from patients' own medication. Documentation of both pre-treatment and post-treatment pain scores occurred in 10% of pain patients. Online Medical Oversight Physician contact occurred during 57 encounters; physician contact was associated with calls in which medication was given (79.3% of calls to physicians received medications), and physician contact was more common when the chief complaint was pain (64.6%) versus breathlessness (53.6%).

## Discussion

This study presents several interesting findings related to differences (and similarities) between the way cancer and non-cancer advanced disease states have been managed by paramedics in the first year following the launch of the Paramedics Providing Palliative Care at Home program, looking at medication use, online physician support, transport, and symptom management for pain and breathlessness in particular.

Previous work suggested that taking a palliative approach might be more comfortable for paramedics when managing a patient with cancer and that the use of opioids for the treatment of breathlessness might be a significant shift in approach [11]. We did find that the same proportion of patients with cancer and non-cancer conditions were treated but likely were undertreated in both instances. Note for instance that 20% of patients with non-cancer illnesses reported dyspnea, while only 10% of them received any treatment. Also, the persistent reliance on salbutamol and ipratropium instead of opioids for dyspnea indicates that, even when treated, the treatment may not have been optimal or in alignment with the palliative care clinical practice guideline.

Paramedics were less likely to consult Medical Oversight Physicians for breathlessness compared to pain and less likely to give medication. The infrequent documentation of post-treatment symptom scores (around 26% of cases) suggests room to improve in monitoring the effectiveness of treatment and titrating accordingly; the potential exists that even those treated did not experience sufficient relief. Calls that were transported were less likely to receive treatment. Patients' own medications were used exclusively or as a supplement to paramedic stock in a significant proportion of calls, and an electronic query is likely to underestimate the treatment given. Even when the cases with patients' own medication are included, these results show there is room for improvement in frequency and quantity of medication administration for pain and breathlessness, and choice of medication for dyspnea in particular. 

There is increasing recognition that patients with non-cancer diagnoses like COPD or CHF benefit from an early and integrated palliative care approach [14-18]. For those with non-cancer advanced diseases such as COPD, symptom exacerbations often result in a trip to the emergency department [19]. Hospitalizations in the non-cancer advanced disease population tend to be intensive, costly to healthcare systems [20] and associated with higher mortality [21]. Acute symptoms, particularly breathlessness, can be very stressful and scary for all patients and their families and caregivers. However, non-cancer advanced disease patients may additionally experience psychological distress perceiving it to be a step towards progressive disability and/or possibly death by suffocation [22], social isolation [23,24], and sets up a cycle of breathlessness which leads to anxiety and fear which creates more breathlessness [25]. However, COPD patients have noted that paramedics in particular provided them with a calming influence during a call for breathlessness [26].

In our study, patients with non-cancer conditions represent an important proportion (60%) of palliative care calls for paramedics. It was not surprising that both groups had similar chief complaints including respiratory distress or breathlessness and pain. Others have found similar symptom burdens between non-cancer advanced diseases and cancer patients [27-30]. Despite pre-launch findings that non-cancer conditions were likely to be undertreated, we found that both groups received similar treatment, although low and may not be optimal. Our results are comparable to an earlier study that found 54% of patients received treatment and 75% were transported by paramedics [2]. The worry of both under- and over-medicating at the end of life has been reported in another study by other health professionals such as nurses [29].

Medication challenges such as access and information by caregivers are common particularly after-hours [30]. Our findings suggest that even with the inclusion of assistance with home medications, the management of pain and breathlessness may not be optimized. Pain scores are a tool available to paramedics to document and assess symptoms pre- and post-treatment. Clinical implications suggest paramedics would benefit from tools/education to increase confidence in applying a palliative approach for non-cancer advanced diseases and in the palliative management of dyspnea. Further research is required to develop useful tools for the assessment of symptom severity following administration of treatment. Paramedics could conduct a more thorough assessment of dyspnea and then manage this symptom in accordance with the severity of presentation. Additional research may also seek to understand the non-cancer palliative care experiences from the point of view of paramedics, as well as differences in outcomes by variables such as level of paramedic practice. 

Limitations

Misclassification of cases may have occurred in some instances. The classification of patients into cancer or non-cancer groups was based on the "Current Diagnosis Category" in SPP, which does not address patients who may have received palliative care following a paramedic call but are not enrolled in the SPP. These patients may have been treated differently or been fundamentally different from those enrolled in SPP and the results of this study may not be generalizable to these individuals. This does not measure non-medication therapy that is provided such as psychosocial support and education to the patient and family and is in addition to any support with home medication which is not electronically queriable.

We also note that there may be potential gaps between practice and documentation due to factors not explored in the present study. Our analysis did not account for specific contextual factors such as rurality, level of certification of responding paramedics, and family support, which may lead to variation in transport outcomes. Sub-analysis is required to determine the demand on the healthcare system and usage in urban vs rural and remote areas across the province. Our methods also only allowed for one chief complaint per visit. It is evident this patient population has complex health problems and often multiple co-morbidities. Some diagnoses may be associated with pain or breathlessness, but the chief complaint may not have been coded to include this.

Work is ongoing to explore the differences in management of different populations requesting a palliative approach to care, and that work has been informed by the learnings, and the limitations, of this project. Some of the limitations of this research will hopefully be addressed by including paramedic as well as patient perspective, collecting socio-demographic variables, as well as broadening data collection beyond one province. This practice may not be generalized directly to other countries where the scope of practice of paramedics is different. 

## Conclusions

This study compares the management of patients with cancer versus non-cancer life-limiting illnesses during the first year of a novel Paramedics and Palliative Care at Home program. The results confirm that there are differences in how paramedics apply a palliative approach in these two cohorts; non-cancer patients are less often likely to achieve non-transport, are less likely to receive medication, and in particular if they are breathless, are more likely to receive usual care instead of a palliative approach. Comfort with applying a palliative approach to non-cancer advanced disease, and in applying palliative therapies to alleviate pain but also particularly dyspnea, will need specific efforts to optimize the impact of programs like this.

## Appendices

**Table 6:** STROBE checklist STROBE: Strengthening the Reporting of Observational Studies in Epidemiology

	Item No.	Recommendation	Page No.	Relevant text from manuscript
Title and abstract	1	(a) Indicate the study’s design with a commonly used term in the title or the abstract	1	Paramedics Providing Palliative Care at Home: A Retrospective Cohort Comparing Symptom Management of Breathlessness and Pain in Cancer Versus Non-Cancer Conditions
		(b) Provide in the abstract an informative and balanced summary of what was done and what was found	1	
Introduction				
Background/rationale	2	Explain the scientific background and rationale for the investigation being reported	1-2	
Objectives	3	State specific objectives, including any prespecified hypotheses	2	Our objective was to describe symptom management and transport outcomes in cancer versus non-cancer advanced diseases, looking specifically at the common symptoms of pain and breathlessness.
Methods				
Study design	4	Present key elements of study design early in the paper	2	We conducted a retrospective cohort study. The paramedic electronic Patient Care Record (ePCR) was queried for all patients with palliative goals of care from July 1, 2015 to June 30, 2016 using patient definitions defined below under "participants." In a sub-analysis, a manual chart review of the 100 most recent consecutive paramedic palliative care responses between February 1, 2016 to June 30, 2016
Setting	5	Describe the setting, locations, and relevant dates, including periods of recruitment, exposure, follow-up, and data collection	2	“study design” and “setting” sub-sections
Participants	6	(a) Cohort study—Give the eligibility criteria, and the sources and methods of selection of participants. Describe methods of follow-up Case-control study—Give the eligibility criteria, and the sources and methods of case ascertainment and control selection. Give the rationale for the choice of cases and controls Cross-sectional study—Give the eligibility criteria, and the sources and methods of selection of participants	2	“participants” sub-section
		(b) Cohort study—For matched studies, give matching criteria and number of exposed and unexposed Case-control study—For matched studies, give matching criteria and the number of controls per case	n/a	
Variables	7	Clearly define all outcomes, exposures, predictors, potential confounders, and effect modifiers. Give diagnostic criteria, if applicable	3	The primary outcome was percent treatment by paramedics while secondary outcomes included transport disposition and treatment efficacy
Data sources/ measurement	8*	For each variable of interest, give sources of data and details of methods of assessment (measurement). Describe comparability of assessment methods if there is more than one group	2	“Data sources” subsection
Bias	9	Describe any efforts to address potential sources of bias		
Study size	10	Explain how the study size was arrived at	2	“study design”, based on time period and then 100 charts for chart review, a convenience sample

Quantitative variables	11	Explain how quantitative variables were handled in the analyses. If applicable, describe which groupings were chosen and why	3	No groupings
Statistical methods	12	(a) Describe all statistical methods, including those used to control for confounding		Results were reported descriptively for the overall population and compared between the cancer and non-cancer groups with n and %, median and interquartile range or mean and standard deviation as appropriate
		(b) Describe any methods used to examine subgroups and interactions		No testing of interactions
		(c) Explain how missing data were addressed		No inferential statistics
		(d) Cohort study—If applicable, explain how loss to follow-up was addressed Case-control study—If applicable, explain how matching of cases and controls was addressed Cross-sectional study—If applicable, describe analytical methods taking account of sampling strategy		Data collection based on one encounter, there is no follow up
		(e) Describe any sensitivity analyses		None
Results				
Participants	13*	(a) Report numbers of individuals at each stage of study—eg numbers potentially eligible, examined for eligibility, confirmed eligible, included in the study, completing follow-up, and analysed	3	995 individuals eligible in chart query, 100 consecutive unique charts for chart review. EHS did not release the non-eligible patient data to us; they would run approximately 100,000 calls per year.
		(b) Give reasons for non-participation at each stage		n/a
		(c) Consider use of a flow diagram		
Descriptive data	14*	(a) Give characteristics of study participants (eg demographic, clinical, social) and information on exposures and potential confounders	3	Table 1
		(b) Indicate number of participants with missing data for each variable of interest	4	Pain scores
		(c) Cohort study—Summarise follow-up time (eg, average and total amount)	3	No follow up
Outcome data	15*	Cohort study—Report numbers of outcome events or summary measures over time		
		Case-control study—Report numbers in each exposure category, or summary measures of exposure		
		Cross-sectional study—Report numbers of outcome events or summary measures	4	Table 2 reports primary outcome, Tables 3 and 4 report secondary outcomes
Main results	16	(a) Give unadjusted estimates and, if applicable, confounder-adjusted estimates and their precision (eg, 95% confidence interval). Make clear which confounders were adjusted for and why they were included		Descriptive statistics only
		(b) Report category boundaries when continuous variables were categorized		
		(c) If relevant, consider translating estimates of relative risk into absolute risk for a meaningful time period		

Other analyses	17	Report other analyses done—eg analyses of subgroups and interactions, and sensitivity analyses		
Discussion				
Key results	18	Summarise key results with reference to study objectives	5	We did find that the same proportion of patients with cancer and non-cancer conditions were treated, but likely were undertreated in both instances. Note for instance that 20% of patients with non-cancer illnesses reported dyspnea, while only 10% of them received any treatment. Also the persistent reliance on salbutamol and ipratropium instead of opioids for dyspnea, indicating that even when treated, the treatment may not have been optimal or in alignment with the palliative care clinical practice guideline. Paramedics were less likely to consult medical oversight physicians for breathlessness compared to pain, and less likely to give medication. Calls that were transported were less likely to receive treatment. Patient’s own medications were used exclusively, or as a supplement to paramedic stock, in a significant proportion of calls, and an electronic query is likely to underestimate treatment given.
Limitations	19	Discuss limitations of the study, taking into account sources of potential bias or imprecision. Discuss both direction and magnitude of any potential bias	6	“limitations” section, many limitations inherent to reliance on clinical documentation
Interpretation	20	Give a cautious overall interpretation of results considering objectives, limitations, multiplicity of analyses, results from similar studies, and other relevant evidence	6	“conclusion”
Generalisability	21	Discuss the generalisability (external validity) of the study results	6	“limitations” section and “conclusion” regarding spread and scale
Other information				
Funding	22	Give the source of funding and the role of the funders for the present study and, if applicable, for the original study on which the present article is based		None, this was an undergraduate medical student project as part of a larger program of research
